# Increased tumor-associated mast cells facilitate thyroid cancer progression by inhibiting CD8^+^ T cell function through galectin-9

**DOI:** 10.1590/1414-431X2023e12370

**Published:** 2023-04-07

**Authors:** Yanli Hou, Qiang Wang, Li Su, Ying Zhu, Yun Xiao, Fei Feng

**Affiliations:** 1 Department of Oncology and Hematology, Chongqing Hospital of Traditional Chinese Medicine, Chongqing, China; 2 Department of Orthodontics, Deyang Stomatological Hospital, Deyang, Sichuan, China

**Keywords:** Mast cells, Thyroid cancer, Stem cell factor, Galectin-9, CD8^+^ T cells

## Abstract

As an important component of solid tumors, mast cells show specific phenotypes in various tumor microenvironments. However, the precise mechanism of mast cell accumulation and the phenotypic features of thyroid cancer (TC) remain largely unknown. Here, we found that mast cells were obviously recruited to tumor tissue by TC-derived stem cell factor (SCF). With tumor progression, mast cell levels increased gradually. In addition, intratumoral mast cells expressed higher levels of the immunosuppressive molecule galectin-9, which effectively suppresses CD8^+^ T-cell antitumor immunity *in vitro.* Blocking galectin-9 on tumor-infiltrating mast cells reversed the immunosuppression of CD8^+^ T cells. In conclusion, our data elucidated novel protumorigenic and immunosuppressive roles of mast cells in TC. In addition, our results indicated that blocking mast cells may impede tumor progression and ameliorate the prognosis of TC patients.

## Introduction

Thyroid cancer (TC) is the most common type of cancer in the endocrine system. In addition, its morbidity and mortality account for approximately 90 and 70% of endocrine malignancies, respectively (1). The majority of TC is differentiated thyroid cancer (DTC), including papillary thyroid cancer (PTC) (>80%), follicular thyroid cancer (FTC) (approximately 3-4%), and medullary thyroid cancer (MTC) (<4%). Anaplastic thyroid carcinoma (ATC) and poorly differentiated thyroid cancer (PDTC) account for less than 10% of TCs (2). Despite valid advancements in prevention and treatment in recent years (3), the pathogenesis of TC has not been well addressed. In the last decade, it has been accepted that the progression and outcome of TC are impacted by the interaction between the tumor and the immune system (4,5). As a crucial role in tumor microenvironments, immune cells can influence cancer prognosis by either fostering or inhibiting tumor initiation, invasion, and metastasis (6). Previous research has focused on the important role of adaptive immunity in TC (7). However, the role of innate immunity in TC progression has rarely been reported.

Mast cells are a type of innate immune cell in the tumor stroma that can release presynthesized soluble mediators stored in the cell or newly synthesized soluble mediators of angiogenesis, tumor stroma reconstruction, and invasiveness (8,9). In addition, mast cells can suppress immune responses against tumors (10). Recently, some limited research on mast cells in TC has mostly focused on the relevance of the infiltration of mast cells in the tumor stroma and overall survival in TC patients in a clinical retrospective study (11), and some other studies have focused on the relationship between the density of mast cell infiltration and angiogenesis (12). The above studies suggest that mast cells are promising as a new therapeutic target for TC. However, the regulatory mechanism and specific effect of mast cells in the TC stroma have yet to be clarified.

Here, we aimed to explore the interactions between mast cells, tumor cells, and CD8^+^ T cells in the TC stroma.

## Material and Methods

### Patients and specimens

Fresh TC tissues (n=113) and autologous non-tumor tissues (n=113) were acquired from TC patients who underwent surgical resection at Chongqing Hospital of Traditional Chinese Medicine. Patients who had received radiation or chemotherapy prior to data collection were excluded. Patients with other primary cancers, infectious diseases, or psychosis were also excluded. The clinical stage of tumors was based on the American Joint Committee on Cancer thyroid cancer TNM classification system (8th edition). The study was discussed and approved by the Ethics Committee of Chongqing Hospital of Traditional Chinese Medicine, and each patient provided written informed consent.

### Flow cytometry

Tumor and non-tumor tissues were washed 3 times with phosphate-buffered saline. Specimens were gathered in RPMI 1640 (containing deoxyribonuclease I (10 mg/mL) and collagenase IV (1 mg/mL)) and digested by a MACS Dissociator (Miltenyi Biotec, Germany). The cell suspension was filtered with a 70-μm cell filter. Mast cells were stained with anti-human CD45, CD117, and FcεRI antibodies. Chemokine receptors on mast cells and mast cell phenotypes were also detected by flow cytometry. The results were analyzed with Flowjo software (Tree Star, USA).

### Isolation and sorting of single-cell suspensions from TC tissues

As noted above, mast cells were labelled with anti-human CD117 and FcεRI antibodies, while CD8^+^ T cells were labelled with anti-human CD8 antibodies, and they were both collected from a single-cell suspension of tumor and autologous non-tumor tissues and sorted by a fluorescence-activating cell sorter (Becton, Dickinson and Company, USA).

### Preparation of TTCS and NTCS

Tumor or autologous non-tumor thyroid tissues were placed in RPMI 1640 to prepare tumor or non-tumor tissue culture supernatants (TTCS or NTCS) for 24 h, and the supernatant was centrifuged (800 *g*, 15 min, 4°C) and harvested.

### Mast cell culture and stimulation

For culture of primary human umbilical cord blood-derived mast cells (hCBMCs), umbilical cord blood was obtained from the Obstetrics and Gynecology Department of Chongqing Hospital of Traditional Chinese Medicine. Umbilical cord blood mononuclear cells were segregated by density gradient centrifugation (1200 *g*, 20 min, 4°C) using Ficoll-Paque Plus (GE Healthcare, USA). CD133^+^ cells were isolated using CD133 microbeads. For the first 6 weeks, CD133^+^ cells were cultured in serum-free expansion medium (StemCell, Canada) supplemented with penicillin (100 U/mL)/streptomycin (100 µg/mL), human recombinant (hr) IL-3 (30 ng/mL, only present during the first 3 weeks), hr IL-6 (50 ng/mL), and stem cell factor (SCF) (100 ng/mL). From week 6, fetal calf serum (FCS) (10%) was added to the culture medium. The generated mast cells were prepared for experiments until week 10. Mast cell purity was determined by surface staining of FcεRI and CD117 and toluidine blue staining.

HMC-1, a mast cell line, was purchased from the China Center for Type Culture Collection (China) and cultured in PRIM1640 (HyClone, USA) containing 10% fetal bovine serum. hCBMCs were triggered with 50% TTCS or autologous 50% NTCS for 24 h, and the expression of galectin-9 on hCBMCs was detected by flow cytometry.

### ELISA

Human thyroid tissues were obtained and homogenized in 0.5 mL of sterile Protein Extraction Reagent (Thermo, USA). The concentrations of SCF in TC and NTCN were identified by ELISA.

### Immunofluorescence

HMC-1 cells were fixed on slides and then triggered by TTCS or NTCS. The cells were washed with PBS, blocked with 20% goat serum for 30 min, and then labelled with galectin-9 and 4,6-diamino-2-phenyl indole (DAPI). Slides were analyzed with a confocal fluorescence microscope (Leica, Germany).

### Chemotaxis assay

For fluorescence-activated cell sorting, 1×10^5^ mast cells from thyroid tumor tissues were placed into the upper chambers (8-μm pore size transwells, Corning, USA). In the lower chambers, 50% TTCS or autologous 50% NTCS was added as the source of chemoattractants. Migration was calculated as the number of cells in the lower chamber after 24 h of culture at 37°C. In some cases, anti-SCF antibody (stem cell factor neutralizing antibody) or control IgG1 (20 μg/mL, IgG1) was added to the TTCS. Human recombinant cytokine SCF (100 ng/mL) and RPMI 1640 medium were added to the lower chambers as positive and blank controls.

### Mast cell/CD8^+^ T cell co-culture system *in vitro*

Mast cells (1×10^5^ cells/well in 96-well plates) segregated from thyroid tumor or non-tumor tissues were co-cultured with autologous thyroid tissue CD8^+^ T cells stained with carboxyfluorescein succinimidyl ester (CFSE) at a 1:2 ratio (mast cell: T cell) in 200 μL RPMI 1640 medium containing 10% fetal bovine serum and human recombinant interleukin (IL)-2 (20 IU/mL) in 96-well plates (coated with 2 μg/mL anti-CD3 and 1 μg/mL anti-CD28 antibodies), with or without a galectin-9 neutralizing antibody (20 μg/mL). Intracellular cytokines in the cells were detected after 5 days of incubation.

### Statistical analysis

The data are reported as means±SE. Student's *t*-test was applied to analyze differences between two groups. When the variances differed, the Mann-Whitney U test was utilized. Multiple comparisons were performed using ANOVA. Pearson correlation analysis and linear regression analysis were used to evaluate the correlations between parameters. SPSS (IBM) was used for all statistical analyses. Data were analyzed using 2-tailed tests, and P<0.05 was considered statistically significant.

## Results

### Increased mast cells in human TC were correlated with tumor progression

**Figure 1:**
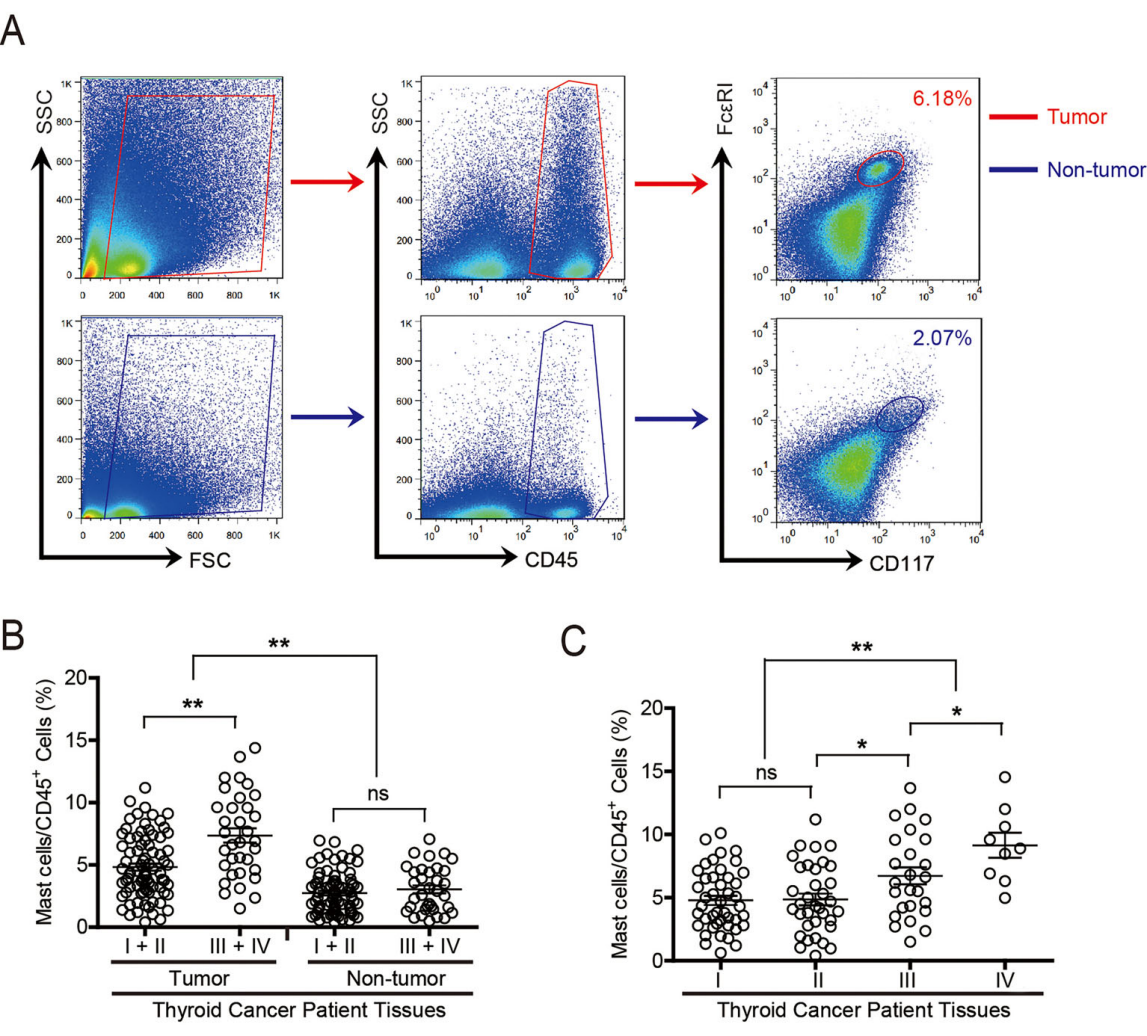
The mast cell percentage was increased in thyroid cancer (TC), and this increase was correlated with disease progression. **A**, Dot plots of molecule labelling for mast cell (CD45^+^ CD117^+^ FcεRI^+^ cells) gating on CD45^+^ cells in tumor and non-tumor tissue of TC patients. **B**, Mast cell percentage in CD45^+^ cells among TNM stages (I+II *vs* III+IV) in tumor and non-tumor tissues of TC patients. The cumulative results of 113 patients with TC are shown. **C**, Intratumoral mast cell percentage among TNM stages is shown. The horizontal bars represent means±SE, and each ring represents 1 patient in Panels B and C. *P<0.05; **P<0.01; (ANOVA). ns: P>0.05.

The percentage of mast cells in tumor tissues was much higher than that in non-tumor tissues of TC patients (Figure 1A). In addition, the accumulation of mast cells in TC at the progressive stage was much higher than that in the early stage (Figure 1B). Furthermore, this intratumoral mast cell infiltration was most significant from stage II onwards (Figure 1C).

These results suggested that mast cells played a protumorigenic role in TC. Consistent with this result, an increased mast cell percentage was correlated with advanced tumor size, tumor (T) stage, and distant metastasis (M) (Supplementary Figure S1). The majority of TC cases were papillary thyroid carcinoma, which has a good prognosis with a 5-year survival rate higher than 90%. Therefore, we did not evaluate the overall survival rates of TC patients. The clinical characteristics of all patients with TC are shown in Table 1. In brief, the above results indicated that increased tumor-associated mast cells were positively associated with TC progression.

**Table 1:** Clinical features of 113 patients with thyroid cancer.

Variables	No. of patients
Gender	
Male	29
Female	84
Age (years)	
<55	64
≥55	49
Tumor size (cm)	
<2	52
≥2	61
Tumor (T) invasion	
T1+T2	79
T3+T4	34
Lymphoid nodal (N) status	
N0+N1	72
N2+N3	41
Distant metastasis (M) status	
M0	105
M1	8
TNM stage	
I+II	79
III+IV	34

### Mast cells were recruited into the tumor by TC-derived SCF

**Figure 2:**
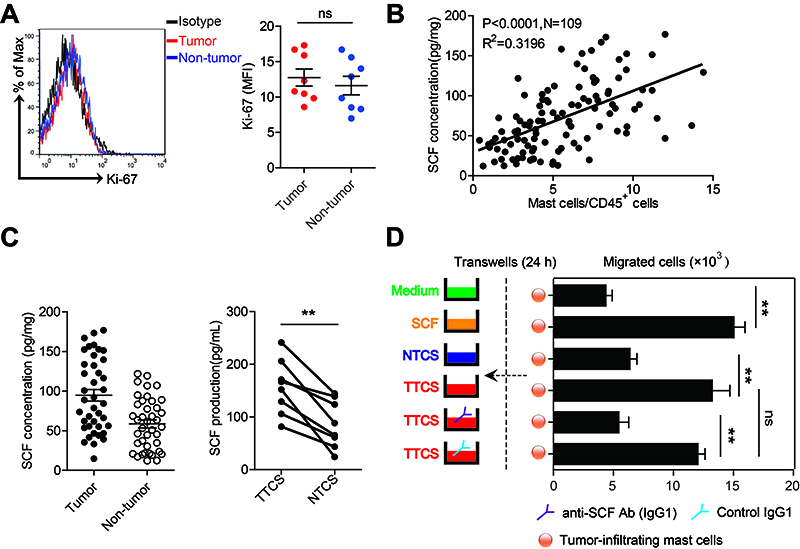
Thyroid cancer (TC)-derived stem cell factor (SCF) mediated mast cell recruitment in TC tissues. **A**, The expression of Ki-67 on mast cells in tumor and non-tumor tissues. Red and blue histograms represent staining of Ki-67 in tumor and non-tumor tissues, respectively; black, isotype control. **B**, The correlations between mast cell percentage in CD45^+^ cell and SCF concentration in TC were detected (n=109). **C**, Differences in SCF levels between tumor tissues and autologous non-tumor tissues and between tumor or non-tumor tissue culture supernatants (TTCS and autologous NTCS) are shown. **D**, Migration of tumor-associated mast cells was detected by transwell assay (n=3). Each ring or dot in Panels A-C represents 1 patient. Data are reported as means±SE. **P<0.01 (ANOVA). ns: P>0.05.

The above results showed that mast cells were increased in the TC microenvironment, so we looked for the reason for this accumulation. We first found that Ki-67 was expressed at low levels in mast cells in the TC microenvironment, suggesting that mast cells had no proliferative activity (Figure 2A). Then, we investigated whether the TC microenvironment could recruit mast cells to the tumor stroma by chemotaxis by screening the chemokine receptors involved in mast cell migration, including CCR2, CCR5, CXCR1, CXCR2, CXCR4, and CXCR7. However, we found that mast cells in the TC microenvironment rarely expressed these common chemokine receptors (Supplementary Figure S2). CD117 (c-kit) is an important marker of mast cells and is also a receptor of SCF. Interestingly, we revealed that the infiltration of mast cells in CD45^+^ leukocytes was positively related to the SCF concentration in thyroid tumor tissue (Figure 2B). In addition, we showed that the concentration of SCF in tumor tissues and TTCS was significantly elevated compared to that in non-tumor tissues and NTCS (Figure 2C).

To elucidate the recruitment of mast cells in tumors by SCF, a mast cell transwell assay was carried out and showed that TTCS markedly recruited more TC-associated mast cells than NTCS from autologous non-tumor tissues, and this effect was blocked by SCF neutralizing antibodies (Figure 2D). We concluded that these data indicated that mast cells were recruited to the thyroid tumor stroma through SCF-mediated chemotaxis.

### Expression of galectin-9 on mast cells was upregulated in TC

**Figure 3:**
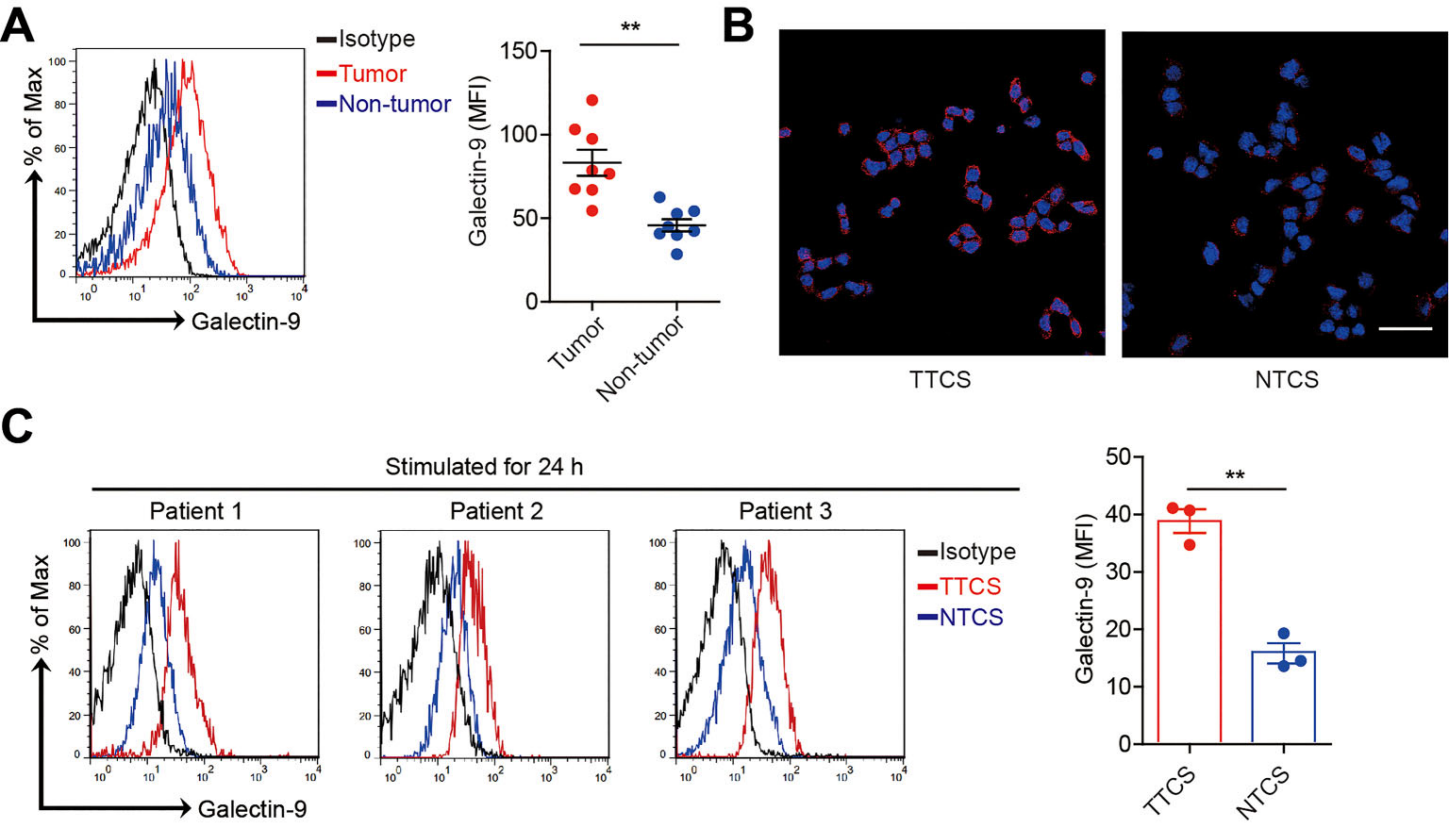
The expression of galectin-9 was upregulated on mast cells in thyroid cancer (TC) tissues. **A**, The expression of galectin-9 on mast cells in TC tumor and non-tumor tissues (n=8). Red and blue histograms represent labelling of galectin-9 in tumor and non-tumor tissues, respectively; black, isotype control. **B**, Expression of galectin-9 on HMC-1 cells (human mast cell line) stimulated by 50% tumor or non-tumor tissue culture supernatants (TTCS and autologous NTCS) for 24 h was detected by immunofluorescence staining. Red, galectin-9; blue, DAPI-stained nuclei. Scale bar: 50 microns. **C**, Expression of galectin-9 on hCBMCs stimulated by 50% TTCS and autologous NTCS for 24 h (n=3). Red and blue histograms represent staining of galectin-9 in tumor and non-tumor tissues, respectively; black, isotype control. Each dot in Panel A represents 1 patient. Data are reported as means±SE. **P<0.01 (*t*-test).

As the phenotype of immune cells often affects their function, we detected the mast cell immune phenotype. Interestingly, we found that the level of the immunosuppressive molecule galectin-9 in tumor-associated mast cells was higher than that in non-tumor mast cells (Figure 3A). However, other immunosuppressive molecules, such as GITRL, CD272, PD-L1, PD-L2, and ICOSL, were expressed on mast cells in tumor and normal tissues without significant differences (Supplementary Figure S3).

Based on the above findings, we hypothesized that the TC microenvironment contributed to the immunosuppressive phenotype of mast cells. Surprisingly, TTCS significantly upregulated galectin-9 expression on mast cells compared to NTCS, which was consistent with our hypothesis (Figure 3B and C). These data indicated that galectin-9 on mast cells may have potential functions in the TC microenvironment.

### Tumor-associated mast cells suppressed CD8^+^ T-cell antitumor immunity by galectin-9

**Figure 4:**
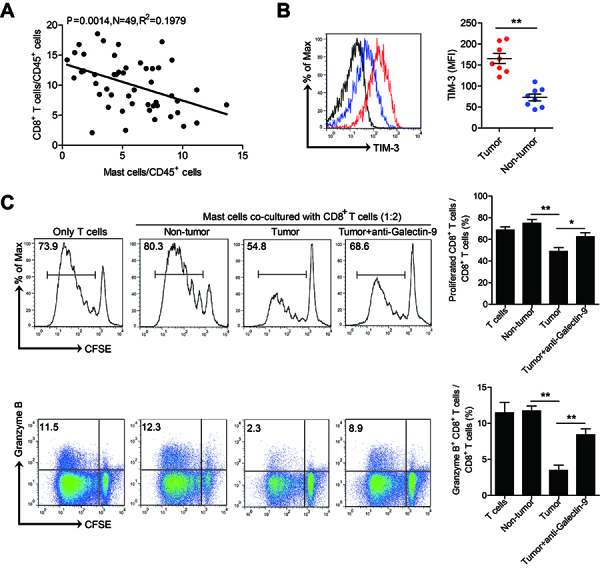
Tumor-associated mast cells suppressed CD8^+^ T-cell antitumor immunity by galectin-9. **A**, The relationship between mast cells and CD8^+^ T cells in thyroid cancer (TC) is shown. **B**, The expression of TIM-3 on CD8^+^ T cells in TC tumor and non-tumor tissues (n=8). Red and blue histograms represent staining of TIM-3 in tumor and non-tumor tissues, respectively; black, isotype control. **C**, Mast cells sorted from tumor or non-tumor tissues were co-cultured with CFSE-labelled autologous peripheral CD8^+^ T cells with or without anti-galectin-9 antibody for 5 days. Representative data and statistical analysis of CD8^+^ T-cell proliferation and granzyme-B secretion are shown as means and SE (n=5). Each dot in Panels A and B represents 1 patient. Data are reported as means±SE. *P<0.05; **P<0.01 (*t*-test or ANOVA). CFSE: carboxyfluorescein succinimidyl ester.

It is well known that CD8^+^ T cells play an important role in antitumor immunity. Therefore, we detected the infiltration of mast cells and CD8^+^ T cells in TC. The results showed a significant negative correlation between the infiltration of mast cells and CD8^+^ T cells in tumor tissue (Figure 4A). In addition, we found that tumor-associated CD8^+^ T cells expressed a higher level of T-cell immunoglobulin domain and mucin domain-3 (TIM-3, the receptor of galectin-9) than non-tumor CD8^+^ T cells (Figure 4B). These results suggested that tumor-infiltrating mast cells might affect the function of CD8^+^ T cells. Therefore, we collected mast cells from tumor and non-tumor tissues of TC patients by fluorescence-activated cell sorting and cultured them with autologous purified peripheral CD8^+^ T cells for 5 days. The co-culture system showed that tumor-infiltrating mast cells had a stronger inhibitory effect on CD8^+^ T-cell proliferation and granzyme-B secretion than non-tumor-derived mast cells (Figure 4C). Furthermore, we added a neutralizing antibody against galectin-9 into a tumor-infiltrating mast cell/CD8^+^ T-cell co-culture system. The results showed that blockade of galectin-9 significantly attenuated CD8^+^ T-cell inhibition mediated by tumor-associated mast cells (Figure 4C), which indicated an immune-suppressive effect of tumor-associated mast cells in tumor immunity. Collectively, these results showed that tumor-infiltrating mast cells suppressed CD8^+^ T-cell antitumor immunity through galectin-9 in TC.

## Discussion

Innate immune cells and adaptive immune cells play considerable roles in the initiation and development of TC (6). The effect of adaptive immune cells in TC has been investigated previously (13,14). Mast cells (a kind of innate immune cell) have been reported in TC (15), but their distribution, phenotype, and functional regulation remain to be clarified. In our study, we showed that mast cells play a protumorigenic role in TC. We showed that mast cells can be recruited to the tumor microenvironment by TC-derived SCF. In addition, the tumor microenvironment efficiently upregulates the expression of galectin-9 on mast cells, which can suppress CD8^+^ T cells in antitumor immunity in a galectin-9-dependent manner in TC, suggesting a novel mechanism of tumor immune escape during TC progression. We analyzed the clinical data and revealed that the infiltration of mast cells in tumor tissue was positively correlated with tumor stage and significantly elevated at advanced stages (III+IV) of TC.

Mast cells are usually involved and recognized in allergic reactions (16). In recent years, some studies have found that mast cell infiltration in the tumor stroma affects cancer progression and prognosis (17,18). Therefore, it is important to elucidate the influence of infiltrating mast cells in TC. We found a significant positive correlation between mast cell infiltration and advanced clinical features of TC, such as tumor size, stage, and distant metastasis (Supplementary Figure 1). Our results suggest that mast cell infiltration in TC could be a promising clinical prognostic marker in the future.

As mast cells were notably increased in tumor tissues, we speculated that intratumoral mast cells either enhanced proliferation or increased migration of mast cells to the tumor microenvironment. We excluded the possibility of the enhanced proliferation of intratumoral mast cells due to the low expression of Ki-67 on mast cells in tumor tissues. Therefore, we predicted that mast cells might be recruited into tumor tissues by chemotaxis. Some previous studies have reported that mast cells express different chemokine receptors, such as CCR2, CCR5, CXCR1, or CXCR4, in different cancers (19-21). Therefore, we screened the expression of chemokine receptors on mast cells, but these chemokine receptors were rarely expressed on mast cells in TC. Recently, a study reported that mast cells could be recruited to the tumor stroma in an SCF-dependent manner (22). The results were consistent with this study; more SCF was secreted in thyroid tumor tissues than in non-tumor tissues, and increased SCF recruited mast cells. This is the first study to show the chemotaxis mechanism of tumor-infiltrating mast cells by SCF in TC.

It is known that the phenotype impacts the function of mast cells. However, the phenotype of tumor-infiltrating mast cells in TC has not been well elucidated. As immunosuppression is a hallmark of cancer (23), we detected mast cells with an immunosuppressive phenotype in TC and revealed that tumor-infiltrating mast cells showed high levels of the immunosuppressive molecule galectin-9. Interestingly, we revealed that intratumoral CD8^+^ T cells expressed high levels of TIM-3 (the receptor of galectin-9). The crosstalk between galectin-9 and TIM-3 is a main mechanism leading to CD8^+^ T cells in immunosuppression (24). At present, some reports have confirmed the role of galectin-9 in cell cycle regulation (25), tumor cell adhesion and angiogenesis (26), metastasis (27), and tumor immune escape (28). In TC, we are the first to show that tumor-associated mast cells exhibit a strong inhibitory role on CD8^+^ T-cell proliferation and granzyme-B secretion in a galectin-9-dependent manner. In addition, tumor-infiltrating mast cells can be triggered to release many biomolecules to induce tumor progression (29). For example, activated mast cells promote tumor growth through tryptase and IL-13 in pancreatic cancer (12).

In short, this study showed a marked protumorigenic role for mast cells in TC and uncovered a novel mechanism by which tumor-infiltrating mast cells intervene in tumor growth via galectin-9. Blocking pathological mast cell accumulation or neutralizing the immunosuppressive effect of galectin-9 on mast cells may be a promising therapeutic strategy for improving the prognosis of TC patients.

## Supplementary Material

Click here to view [pdf].

## References

[1] Lim H, Devesa SS, Sosa JA, Check D, Kitahara CM (2017). Trends in thyroid cancer incidence and mortality in the United States, 1974-2013. JAMA.

[2] Carling T, Udelsman R (2014). Thyroid cancer. Ann Rev Med.

[3] Luster M, Weber T, Verburg FA (2014). Differentiated thyroid cancer-personalized therapies to prevent overtreatment. Nat Rev Endocrinol.

[4] Ferrari SM, Fallahi P, Galdiero MR, Riffilli I, Elia G, Ragusa F (2019). Immune and inflammatory cells in thyroid cancer microenvironment. Int J Mol Sci.

[5] Liotti F, Prevete N, Vecchio G, Melillo RM (2019). Recent advances in understanding immune phenotypes of thyroid carcinomas: prognostication and emerging therapies. F1000Res.

[6] Klemm F, Joyce JA (2015). Microenvironmental regulation of therapeutic response in cancer. Trends Cell Biol.

[7] French JD, Kotnis GR, Said S, Raeburn CD, Mclntyre RC, Klopper JP (2012). Programmed death-1+ T cells and regulatory T cells are enriched in tumor-involved lymph nodes and associated with aggressive features in papillary thyroid cancer. J Clin Endocrinol Metab.

[8] Melillo RM, Guarino V, Avilla E, Galdiero MR, Liotti F, Prevete N (2010). Mast cells have a protumorigenic role in human thyroid cancer. Oncogene.

[9] Sammarco G, Gadaleta CD, Zuccala V, Albayrak E, Patruno R, Milella P (2018). Tumor-associated macrophages and mast cells positive to tryptase are correlated with angiogenesis in surgically-treated gastric cancer patients. Int J Mol Sci.

[10] Wasiuk A, Dalton DK, Schpero WL, Stan RV, Conejo-Garcia JR, Noelle RJ (2012). Mast cells impair the development of protective anti-tumor immunity. Cancer Immunol Immunother.

[11] Hu G, Wang S, Cheng P (2018). Tumor-infiltrating tryptase(+) mast cells predict unfavorable clinical outcome in solid tumors. Int J Cancer.

[12] Sammarco G, Varricchi G, Ferraro V, Ammendola M, De Fazio M, Altomare DF (2019). Mast cells, angiogenesis and lymphangiogenesis in human gastric cancer. Int J Mol Sci.

[13] Cunha LL, Marcello MA, Nonogaki S, Morari EC, Soares FA, Vassallo J (2015). CD8+ tumour-infiltrating lymphocytes and COX2 expression may predict relapse in differentiated thyroid cancer. Clin Endocrinol (Oxf).

[14] Cunha LL, Morari EC, Guihen AC, Razolli D, Gerhard R, Nonogaki S (2012). Infiltration of a mixture of immune cells may be related to good prognosis in patients with differentiated thyroid carcinoma. Clin Endocrinol (Oxf).

[15] Visciano C, Liotti F, Prevete N, Cali G, Collina F, de Paulis A (2015). Mast cells induce epithelial-to-mesenchymal transition and stem cell features in human thyroid cancer cells through an IL-8-Akt-Slug pathway. Oncogene.

[16] Galli SJ, Tsai M (2012). IgE and mast cells in allergic disease. Nat Med.

[17] Franco G, Guarnotta C, Frossi B, Piccaluga PP, Boveri E, Gulino A (2014). Bone marrow stroma CD40 expression correlates with inflammatory mast cell infiltration and disease progression in splenic marginal zone lymphoma. Blood.

[18] Giannou AD, Marazioti A, Spella M, Kanellakis NI, Apostolopoulou H, Psallidas I (2015). Mast cells mediate malignant pleural effusion formation. J Clin Investig.

[19] Bodduluri SR, Mathis S, Maturu P, Krishnan E, Satpathy SR, Chilton PM (2018). Mast cell-dependent CD8(+) t-cell recruitment mediates immune surveillance of intestinal tumors in Apc(min/+) mice. Cancer Immunol Res.

[B20] Lv Y, Zhao Y, Wang X, Chen N, Mao F, Teng Y (2019). Increased intratumoral mast cells foster immune suppression and gastric cancer progression through TNF-α-PD-L1 pathway. J Immunother Cancer.

[21] Varricchi G, Galdiero MR, Loffredo S, Marone G, Iannonne R, Marone G (2017). Are mast cells MASTers in cancer?. Front Immunol.

[22] Huang B, Lei Z, Zhang GM, Li D, Song C, Li B (2008). SCF-mediated mast cell infiltration and activation exacerbate the inflammation and immunosuppression in tumor microenvironment. Blood.

[23] Schreiber RD, Old LJ, Smyth MJ (2011). Cancer immunoediting: integrating immunity's roles in cancer suppression and promotion. Science.

[24] Meggyes M, Miko E, Polgar B (2014). Peripheral blood TIM-3 positive NK and CD8+ T cells throughout pregnancy: TIM-3/galectin-9 interaction and its possible role during pregnancy. PLoS One.

[25] Zhu C, Anderson AC, Schubart A, Xiong H, Imitola J, Khoury SJ (2005). The Tim-3 ligand galectin-9 negatively regulates T helper type 1 immunity. Nat Immunol.

[26] Kageshita T, Kashio Y, Yamauchi A, Seki M, Abedin MJ, Nishi N (2002). Possible role of galectin-9 in cell aggregation and apoptosis of human melanoma cell lines and its clinical significance. Int J Cancer.

[27] Fainaru O, Almog N, Yung CW, Nakai K, Montoya-Zavala, Abdollahi A (2010). Tumor growth and angiogenesis are dependent on the presence of immature dendritic cells. FASEB J.

[28] Seki M, Oomizu S, Sakata KM, Sakata A, Arikawa T, Watanabe K (2008). Galectin-9 suppresses the generation of Th17, promotes the induction of regulatory T cells, and regulates experimental autoimmune arthritis. Clin Immunol.

[29] Marichal T, Tsai M, Galli SJ (2013). Mast cells: potential positive and negative roles in tumor biology. Cancer Immunol Res.

